# Interleukin-27 remodels the bone marrow niche to suppress B-cell development and leukaemia progression in mouse models

**DOI:** 10.1016/j.ebiom.2026.106239

**Published:** 2026-04-02

**Authors:** Jian-Min Zhu, Jing Xia, Kai-Ming Chen, Xue-Feng Bai, Cai-Wen Duan

**Affiliations:** a Pediatric Translational Medicine Institute, Shanghai Children's Medical Center, Shanghai Jiao Tong University School of Medicine, Shanghai, 200127, China; b Faculty of Pharmaceutical Sciences, Shenzhen University of Advanced Technology, Shenzhen, Guangdong, 518107, China

**Keywords:** IL-27, BM microenvironment, B-cell development, B-ALL, Haematopoiesis

## Abstract

**Background:**

Interleukin-27 (IL-27) is an immunoregulatory cytokine, but its role in B-cell haematopoiesis and B-cell acute lymphoblastic leukaemia (B-ALL) within the bone marrow (BM) niche remains unclear.

**Methods:**

IL-27 was delivered in vivo using adeno-associated virus. B-cell reconstitution, mixed BM chimeras, and an N-myc-driven B-ALL model were analysed by flow cytometry, transcriptional profiling, and survival studies. Group comparisons were assessed using Student's t-test, and survival was evaluated by Kaplan–Meier analysis with log-rank tests.

**Findings:**

Sustained IL-27 expression selectively impaired B-cell reconstitution while preserving overall haematopoietic recovery, with marked reductions in CLPs and early B-cell subsets. IL-27 directly inhibited early B-lineage differentiation and concurrently remodelled the BM microenvironment by downregulating VCAM-1, ICAM-2, CXCL12, and IGF-1. These niche alterations were associated with reduced BM-resident B-ALL burden, enhanced chemotherapy efficacy, and improved survival in B-ALL-bearing mice. IL-27 showed no direct cytotoxicity toward B-ALL cells, supporting an indirect, niche-mediated mechanism.

**Interpretation:**

IL-27 constrains B-cell haematopoiesis and B-ALL progression through coordinated progenitor inhibition and BM niche remodelling, revealing a cytokine-driven strategy with the potential to enhance leukaemia therapy.

**Funding:**

This work was supported by the 10.13039/501100012166National Key R&D Program of China (Grant No. 2021YFA1100800 to A.-B.L.) and the 10.13039/501100001809National Natural Science Foundation of China (Grant No. 82100180).

**Research in context**

**Evidence before this study**

Prior studies have shown that IL-27 broadly regulates immune cells, particularly T cells and myeloid cells, but its role in B-cell progenitors and leukaemia-supportive BM niches remains unclear. Most evidence is derived from in vitro or isolated cell models, with limited data on how cytokines regulate progenitors and remodel the niche in vivo.

**Added value of this study**

Using in vivo models, we demonstrate that IL-27 regulates B-cell development and B-ALL progression through dual mechanisms: acting on early B-cell progenitors, particularly CLPs, and remodelling the BM microenvironment to reduce niche support, including key factors such as VCAM-1, CXCL12, and IGF-1. We further show that IL-27-mediated niche alterations enhance the efficacy of chemotherapy, with additional benefits observed in CAR-T-based B-ALL models.

**Implications of all the available evidence**

These findings suggest that IL-27 could complement existing therapies by limiting leukaemic survival and restraining B-cell development. Mechanistically, they highlight how cytokine-driven niche remodelling influences both normal B-cell haematopoiesis and leukaemia progression, with potential relevance for human B-cell malignancies and immune-mediated disorders.

## Introduction

Interleukin-27 (IL-27) is a heterodimeric cytokine of the IL-6/IL-12 family, composed of the p28 and Epstein–Barr virus-induced gene 3 (EBI3) subunits.^1^ IL-27 signals through a receptor complex formed by IL-27Rα (WSX-1) and gp130 and exerts broad immunoregulatory functions.^2^^,^^3^ Recent studies highlight its crucial role in sustaining T-cell responses under chronic antigen exposure, including in tumours and persistent viral infections.^4^, ^5^, ^6^, ^7^ In addition, IL-27 has been implicated in regulating haematopoietic stem and progenitor cell activity, limiting differentiation and protecting against inflammatory stress-induced exhaustion of the HSPC pool, suggesting potential effects on early haematopoiesis.^8^ Our previous work has also demonstrated therapeutic benefits of IL-27 in diverse disease models, including cancer,^9^, ^10^, ^11^, ^12^, ^13^ experimental autoimmune encephalomyelitis,^14^ colitis,^15^ scurfy disease^16^ and graft-versus-host disease.^17^

Beyond its established effects on T cells, IL-27 is increasingly recognised as a regulator of B-cell biology. The IL-27 receptor is constitutively expressed on naïve and memory B cells and is further upregulated on germinal centre B cells following CD40 stimulation.^18^ In Epstein–Barr virus (EBV)-driven transformation, IL-27 promotes B-cell survival via IL-27Rα.^19^ Within germinal centres, IL-27 enhances IL-21 production and supports T follicular helper (Tfh) cell functions, which are essential for antibody responses.^20^^,^^21^ Conversely, studies in SIV-infected macaques show that IL-27 skews Tfh differentiation towards a Th1-like programme, impairing germinal centre responses and reducing B-cell immunity.^22^ Furthermore, IL-27 has been reported to inhibit paediatric B-ALL proliferation by inducing apoptosis.^23^ These context-dependent findings underscore the complexity of IL-27's role in B-cell regulation and highlight the need for further mechanistic investigation.

B-cell development occurs in the bone marrow (BM), where haematopoietic stem cells differentiate into common lymphoid progenitors (CLPs) and progress through pre-pro-B, pro-B, pre-B, and immature B-cell stages, each defined by distinct surface markers and transcriptional programmes.^24^ The BM microenvironment provides a specialised niche that regulates this process via stromal cells, extracellular matrix, cytokines, and adhesion molecules.^25^, ^26^, ^27^, ^28^, ^29^ For example, α4β1 integrins on B-cell progenitors interact with VCAM-1 on stromal cells to ensure retention and survival.^30^ CXCL12 secreted by BM stromal cells orchestrates B-cell migration and differentiation via CXCR4,^31^ while IGF-1 derived from mesenchymal stromal cells (MSCs) facilitates the pro-to pre-B-cell transition.^32^ Disruptions in these tightly regulated interactions can result in immunodeficiency, autoimmunity, or malignant transformation such as B-ALL.^33^

Importantly, the BM niche not only supports normal B-cell development but also protects leukaemic cells by providing survival signals and mediating adhesion-dependent chemoresistance.^25^ CXCL12 signalling via CXCR4 has been implicated in leukaemia progression, while blockade of adhesion molecules such as VLA-4 can reverse MSC-conferred chemoresistance.^34^^,^^35^ Additionally, inhibition of IGF1R markedly restricts leukaemia progression.^36^

Despite extensive evidence for IL-27's immunomodulatory functions, its precise role in B-cell development within the BM niche remains poorly understood. In particular, whether IL-27 can remodel the BM microenvironment to influence B-ALL survival has not been systematically addressed.

Here, we investigate the effects of exogenous IL-27 on B-cell development and leukaemogenesis. We show that IL-27 acts through dual mechanisms: acting on CLPs and remodelling the BM niche, thereby diminishing support for both normal B cells and B-ALL. These findings identify IL-27 as a regulator of B-cell development and function, with potential implications for B-cell malignancies and immune-mediated disorders.

## Methods

### Mice

Mice on a C57BL/6 genetic background, including wild-type (WT, CD45.2^+^; SM-001, Shanghai Model Organisms), CD45.1^+^ (RRID:IMSR_NM-KI-210226), IL-27R^fl/fl^ (RRID:IMSR_NM-CKO-2112768), Lyz2^Cre^ (RRID:IMSR_JAX:018956), CD4^Cre^ (RRID:IMSR_JAX:022071), CD19^Cre^ (RRID:IMSR_JAX:006785), IL-27R^−/−^ (RRID:IMSR_GPT:T005577) and Rag1^−/−^ (RRID:IMSR_GPT:T004753) mice, as well as NSG (RRID:IMSR_NM-NSG-001) mice were maintained under specific pathogen-free (SPF) conditions at the animal facility of Shanghai Children's Medical Center. Mice were used at 6–10 weeks of age. IL-27R^fl/fl^ mice were crossed with Lyz2^Cre^, CD4^Cre^, or CD19^Cre^ mice to generate lineage-specific IL-27R deficient mice where indicated. Both male and female mice were used, with sex matched within each experiment. Littermates were randomly assigned to experimental groups. A total of 233 mice were used for all experiments in this study.

### Recombinant AAV production and In Vivo IL-27 delivery

IL-27 cDNA encoding EBI3 and p28 linked by a flexible peptide (VPGVGVPGVG) was cloned into an AAV backbone under the CMV–chicken β-actin (CAG) promoter, as described previously.^12^, ^13^, ^14^, ^15^, ^16^, ^17^ Recombinant AAV2/8 vectors expressing murine IL-27 (AAV-IL-27), human IL-27 (AAV-hIL-27), or empty controls (AAV-Ctrl) were produced and purified by EKBioTech (Shanghai, China). Mice were injected intramuscularly with 1 × 10^11^ vg per animal (50 μL per site, two sites).

### Bone marrow transplantation and chimera generation

Recipient mice were lethally irradiated (7.5 Gy total body γ-irradiation) to ablate endogenous haematopoiesis, followed by intravenous transplantation of donor BM cells (2 × 10^6^ cells per mouse) within 24 h.

For mixed BM chimera experiments, donor BM cells from CD45.1^+^ WT mice and IL-27R^−/−^ (CD45.1^−^) mice were mixed 1:1 prior to transplantation. Haematopoietic chimaerism and immune reconstitution were analysed at 5 and 10 weeks post-transplant.

### B-ALL mouse model

BM cells from N-myc-driven B-ALL mice, previously serially passaged in vivo, were cryopreserved in liquid nitrogen. Thawed BM cells (1 × 10^5^ per mouse) were intravenously injected to induce aggressive leukaemia within 10–12 days. Detailed model generation and characterisation have been described previously.^37^^,^^38^

### Flow cytometry and cell subset definition

Single-cell suspensions from BM and spleen were prepared using standard protocols. Cells were stained with fluorochrome-conjugated antibodies sourced from eBioscience, BD Biosciences, and BioLegend, all of which were validated by the manufacturers for flow cytometry applications. Detailed information, including clone names, catalogue numbers, and RRID tags for all antibodies, is provided in the Supplementary Table S1. To ensure staining specificity and optimal signal-to-noise ratios, all antibodies were titrated in-house, and appropriate isotype controls or Fluorescence Minus One controls were employed for gating. Flow cytometry was performed on BD Canto or Beckman CytoFLEX instruments, and data were analysed using FlowJo software (V10.5.3).

B-cell developmental stages were defined as follows: Pre-pro-B: B220^+^CD19^−^CD43^hi^IgM^−^; Pro-B: CD19^+^B220^+^CD43^hi^IgM^−^; Pre-B: CD19^+^B220^+^CD43^low^IgM^−^; Immature B: CD19^+^B220^+^IgM^+^; CLP: Lin^−^c-Kit^low^Sca-1^low^CD127^+^Flt3^+^α4β7^−^.

### Quantitative PCR

Total RNA was extracted from BM cells, sorted populations, or cultured cells using TRIzol (15596018CN, ThermoFisher). cDNA synthesis was performed using a reverse transcription kit (TCH026, Takara). qPCR was conducted using SYBR Green Master Mix (330520, QIAGEN) on a CFX Opus 96 system. Gene expression was normalised to β-actin and analysed using the ΔΔCt method.

### In Vitro stimulation of Reh cells

The human B-ALL cell line Reh (RRID:CVCL_1650; kindly provided by Professor Bin-Bing Stephen Zhou, SCMC) was cultured in RPMI 1640 (C11875500CP, ThermoFisher) with 10% FBS (BVS500, BIOVISION). The Reh cell line was authenticated by short tandem repeat (STR) profiling by Shanghai BioWing. Cells were routinely tested and confirmed to be free of mycoplasma contamination. Cells were treated with recombinant human IL-27 (50 ng/mL; 10076-H08S, Sino Biological) and/or decitabine (DAC, 0.25 μM; HY-A0004, MedChemExpress) for 72 h prior to RNA extraction and qPCR.

### Mesenchymal stromal cell culture

Mouse and human BM MSCs were isolated and cultured as previously described.^39^^,^^40^ Murine MSCs were maintained in MesenCult basal medium (05513, STEMCELL Technologies). Human MSCs were derived from paediatric BM samples collected during cardiac surgery, isolated by density gradient centrifugation, and cultured in MSC basal medium (HUXMA-90011, Cyagen). Cells were treated with recombinant murine IL-27 (51107-M08H, Sino Biological) or human IL-27 (50 ng/mL) for 24 h prior to RNA extraction.

### RNA sequencing and bioinformatic analysis

RNA-seq libraries were prepared from 1 × 10^6^ cells per sample and sequenced by BGI and Oebiotech. Expression was quantified as TPM or FPKM, as indicated in the corresponding figures. Immune cell composition and B-cell subset proportions were inferred using CIBERSORTx with reference matrices from published scRNA-seq datasets (GSE168158, GSE186158).^41^^,^^42^ Parameters: perm = 1000, QN = TRUE. GSVA and GO enrichment analyses were performed in R (v4.3.1); data visualisation used ggplot2 and pheatmap. Raw RNA sequencing data have been deposited in the Gene Expression Omnibus (GEO) under accession numbers GSE320585 (splenic B cells, BM cells, BM B-ALL cells and human MSCs) and GSE227476 (BM Ly6G^+^ cells).

### CAR-T cell therapy and chemotherapy

Murine CAR-T cells were generated as described previously.^43^ T cells were isolated from inguinal lymph nodes and activated with plate-bound anti-CD3 (100340, BioLegend) and anti-CD28 (102116, BioLegend). Cells were cultured in RPMI 1640 supplemented with 10% FBS, 1% penicillin–streptomycin, 50 μM 2-mercaptoethanol (31350010, Gibco), 1% L-alanyl-l-glutamine (E607060, BBI-lifesciences), 1% non-essential amino acids (11140050, Gibco), 1% HEPES (E607018, BBI-lifesciences), and 300 IU/mL recombinant human IL-2 (TBD20-2, TDB science), then transduced with murine CD19 CAR retroviral vectors (MSGV-1D3-28z; P44010, MiaoLing Plasmid). CAR expression was confirmed prior to adoptive transfer. Mice received cyclophosphamide (CTX, 4.5 mg; S30563, YuanYe Bio-Technology) and fludarabine (FLU, 0.75 mg; S67909, YuanYe Bio-Technology) at full or half doses as indicated. Survival was monitored longitudinally.

### Human G-PBSC transplantation in NSG mice

NSG mice were subjected to intravenous transplantation of human G-PBSCs (5 × 10^7^ cells per mouse) following sublethal γ-irradiation (1.5 Gy). Mice were treated with AAV-Ctrl or AAV-hIL-27, and human B-cell reconstitution was analysed by flow cytometry.

### Statistics

GraphPad Prism (GraphPad Software) was used for statistical analyses. Data normality was assessed using the Shapiro–Wilk test where sample size permitted. For datasets without significant deviation from normality (P > 0.05), two-tailed unpaired Student's t-tests were used for comparisons between two groups.

For experiments with very limited sample sizes, formal statistical testing was not performed, and results were interpreted descriptively. Sample sizes for all experiments are indicated in the corresponding figures or figure legends.

Each dot in scatter plots represents an individual biological replicate. Survival analyses were performed using Kaplan–Meier curves with log-rank tests. Data are presented as mean ± SD where applicable. A P value <0.05 was considered statistically significant and is denoted as ∗ (P < 0.05), ∗∗ (P < 0.01), ∗∗∗ (P < 0.001), and ns (P > 0.05). Exact P values are provided where indicated.

As many experiments involved exploratory mechanistic animal studies, formal a priori power calculations were not performed due to the lack of reliable prior estimates of variance and effect size. Sample size adequacy was therefore guided by the resource equation method (E = N − G; ideal range 10–20) as previously described.^44^ Key findings were supported by independent animal experiments and complementary experimental systems.

### Ethics statement

Human BM samples and G-PBSCs were obtained with informed consent and approved by the Institutional Review Board of Shanghai Children's Medical Center (Approval No. SCMCIRB-K2024163-1).

All animal experiments were reviewed and approved by the Laboratory Animal Welfare and Ethics Committee of Shanghai Children's Medical Center (Approval No. SCMC-LAWEC-2022-016). All procedures were performed in accordance with institutional guidelines and the ARRIVE guidelines.

### Role of funders

The funder provided financial support following project approval and required submission of progress and final reports, but played no role in the study design, conduct, analysis, or reporting.

## Results

### Exogenous IL-27 impairs early B-cell development in the bone marrow

Consistent with our previous observations, AAV-mediated IL-27 delivery markedly reduced the proportion of B cells among tumour-infiltrating lymphocytes.^12^ To determine whether this reflected impaired B-cell development rather than altered peripheral expansion, we analysed splenic and BM B-cell compartments following systemic IL-27 exposure.

**Fig. 1:**
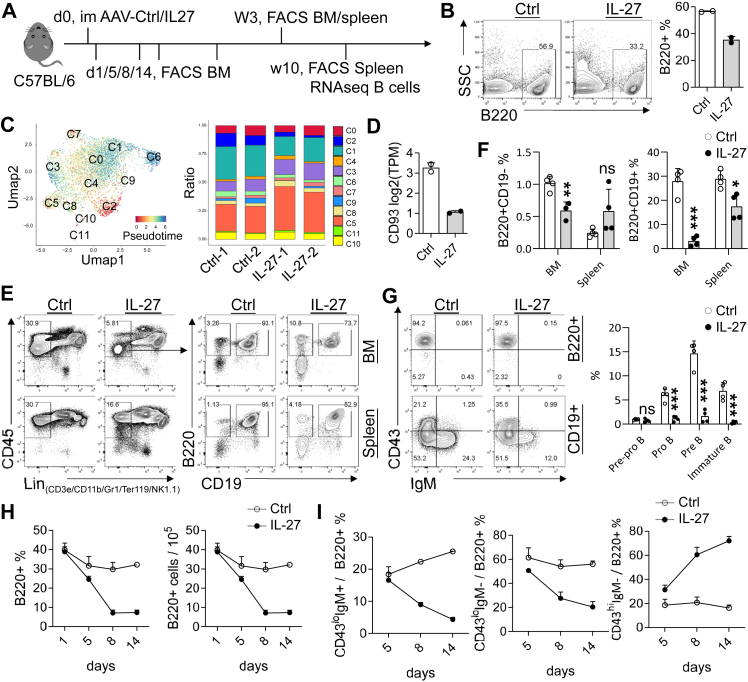
**IL-27 impairs early B-cell development in the bone marrow.** (A) Experimental scheme for AAV-mediated IL-27 delivery and analysis of splenic and BM B-cell compartments. (B–D) Splenic B-cell analysis 10 weeks after AAV-Ctrl or AAV-IL-27 treatment (n = 2 per group). (B) Frequency of total splenic B cells. (C) CIBERSORTx-based deconvolution of splenic B-cell subsets, including clustering and pseudotime analysis (left) and relative subset distribution (right). (D) *Cd93* expression (TPM) in splenic B cells. (E–G) Analysis of splenic and BM B-cell compartments 3 weeks after treatment (n = 4 per group). (E) Representative flow cytometry plots. (F) Frequencies of B220^+^CD19^−^ and B220^−^CD19^+^ populations in the BM and spleen. (G) Relative distribution of B-cell developmental subsets in the BM. (H–I) Kinetics of B-cell populations following IL-27 treatment (n = 2 per group at each time point). (H) Frequency and absolute number of BM B220^+^ B cells. (I) Proportions of B-cell subsets within the B220^+^ compartment at the indicated time points. ∗P < 0.05, ∗∗P < 0.01, ∗∗∗P < 0.001; ns, not significant; unpaired Student's t-test.

IL-27 treatment was associated with a reduced proportion of total splenic B cells at 10 weeks post-exposure (Fig. 1A and B). Transcriptomic deconvolution using CIBERSORTx indicated a relative decrease in early B-cell–associated signatures, which was supported by clustering analyses (Fig. 1C). Consistently, expression of the immature B-cell marker Cd93 appeared reduced (Fig. 1D), suggesting that IL-27 may preferentially influence early B-cell differentiation rather than selectively depleting mature B cells.

We next examined the BM compartment. IL-27 caused a pronounced reduction in BM B220^+^ B cells (Fig. 1E and F). The majority of B220^+^CD19^-^ cells in the BM exhibited a pre-pro-B phenotype, whose relative proportion remained largely unchanged following IL-27 treatment. In contrast, downstream B-cell subsets—including pro-B, pre-B, and immature B cells—were markedly reduced upon IL-27 exposure (Fig. 1G).

Kinetic analysis suggested that BM B-cell loss became apparent by day 8 and was accompanied by an increase in early B-cell progenitors (B220^+^CD43^hi^IgM^−^) (Fig. 1H and I), consistent with a potential early developmental blockade.

Together, these findings demonstrate that exogenous IL-27 selectively impairs early B-cell development in the BM, leading to reduced B-cell output in peripheral lymphoid organs.

### Exogenous IL-27 reshapes immune reconstitution after bone marrow transplantation

**Fig. 2:**
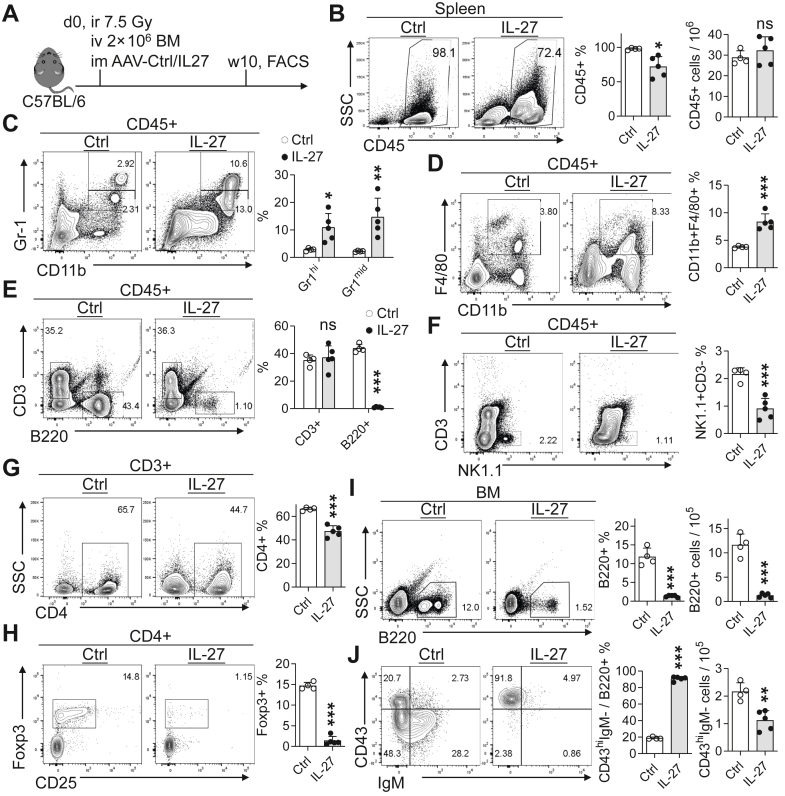
**Exogenous IL-27 reshapes immune reconstitution after bone marrow transplantation.** (A) Experimental scheme. WT mice underwent BM transplantation followed by AAV-Ctrl or AAV-IL-27 treatment. Splenic and BM immune reconstitution was analysed at 10 weeks after treatment (n = 4 mice for the Ctrl group; n = 5 mice for the IL-27 group). (B) Percentages and absolute numbers of CD45^+^ splenocytes. (C–F) Frequencies of major immune cell populations within CD45^+^ splenocytes: myeloid cells (C), macrophages (D), T and B cells (E), and NK cells (F). (G) Frequency of CD4^+^ T cells among splenic T cells. (H) Proportion of Tregs among CD4^+^ T cells. (I–J) BM B-cell reconstitution. Frequency of BM B220^+^ B cells (I) and distribution of B220^+^ B-cell subsets (J). ∗P < 0.05, ∗∗P < 0.01, ∗∗∗P < 0.001; ns, not significant; unpaired Student's t-test.

Given that IL-27 selectively impaired early B-cell development under steady-state conditions, we next assessed its impact on immune reconstitution after BM transplantation. Despite sustained IL-27 exposure, overall haematopoietic recovery, as assessed by total CD45^+^ cell reconstitution, remained largely intact (Fig. 2A and B).

In contrast to preserved global reconstitution, IL-27 markedly altered immune composition. Myeloid cells, including macrophages, were significantly expanded within the CD45^+^ compartment (Fig. 2C and D), indicating a skewing towards myelopoiesis. Conversely, lymphoid reconstitution was selectively impaired: splenic B cells were profoundly reduced (Fig. 2E), while NK cells showed a modest decrease (Fig. 2F). Total T-cell frequencies were largely maintained; however, the proportion of CD4^+^ T cells was reduced, accompanied by a marked decrease in Tregs within the CD4^+^ compartment (Fig. 2G and H).

Consistent with defective peripheral B-cell recovery, the BM compartment exhibited a pronounced reduction in both the frequency and absolute number of B220^+^ B cells in IL-27-treated mice (Fig. 2I). Analysis of BM B-cell subsets revealed a disrupted developmental distribution, characterised by relative enrichment of early B-cell progenitors and marked depletion of downstream differentiated B-cell subsets (Fig. 2J), consistent with an early developmental blockade.

Collectively, these findings indicate that exogenous IL-27 does not compromise global haematopoietic recovery post-transplant but selectively reshapes immune reconstitution, favouring myeloid output while impairing B-cell recovery and modulating specific lymphoid subsets.

### Exogenous IL-27 suppresses CLP formation and disrupts early B-lineage commitment in the bone marrow

**Fig. 3:**
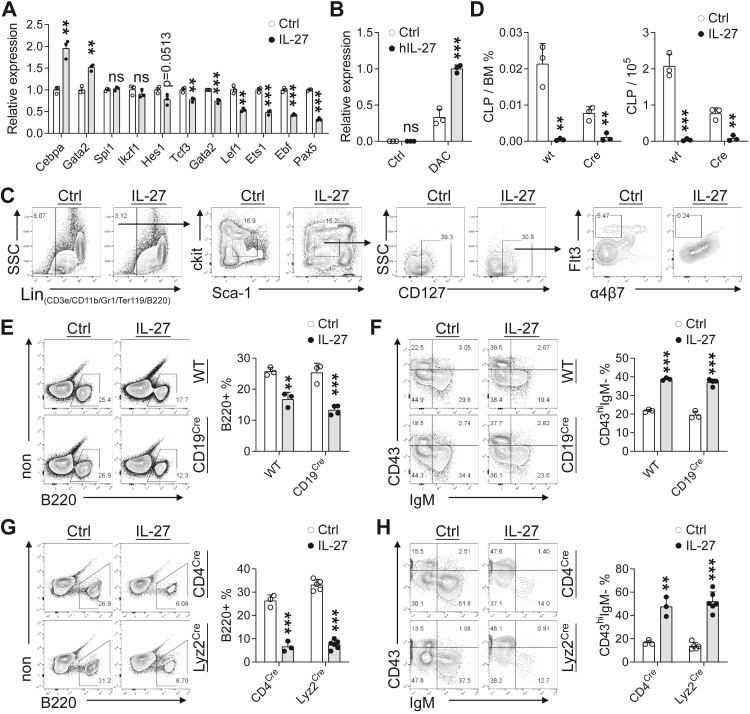
**Exogenous IL-27 inhibits CLP formation in the bone marrow.** (A) qPCR analysis of B-lineage- and myeloid-associated transcription factors in BM cells 5 days after AAV-Ctrl or AAV-IL-27 administration (n = 3 per group). (B) qPCR analysis of *CEBPA* expression in Reh cells treated with hIL-27 and DAC for 3 days (n = 3 per group). (C–D) Frequencies and absolute numbers of CLPs in WT and CD19^Cre^;IL-27R^fl/fl^ mice (n = 3 per group). (E–F) BM B-cell frequency (E) and proportion of B220^+^CD43^hi^IgM^−^ B cells (F) in WT and CD19^Cre^;IL-27R^fl/fl^ mice 2 weeks after AAV treatment (n = 3 for WT Ctrl group; n = 3 for WT IL-27 group; n = 3 for CD19^Cre^ Ctrl group; n = 4 for CD19^Cre^ IL-27 group). (G–H) BM B-cell frequency (G) and proportion of B220^+^CD43^hi^IgM^−^ B cells (H) in CD4^Cre^;IL-27R^fl/fl^ and Lyz2^Cre^;IL-27R^fl/fl^ mice 2 weeks after AAV treatment (n = 3 for CD4^Cre^ Ctrl group; n = 3 for CD4^Cre^ IL-27 group; n = 5 for Lyz2^Cre^ Ctrl group; n = 6 for Lyz2^Cre^ IL-27 group). ∗P < 0.05, ∗∗P < 0.01, ∗∗∗P < 0.001; ns, not significant; unpaired Student's t-test.

To investigate the mechanisms underlying IL-27-mediated inhibition of early B-cell development, we first examined transcriptional programmes in the BM following IL-27 exposure. IL-27 induced coordinated repression of key B-lineage-associated transcription factors, including *Tcf3* (E2A), *Lef1*, *Ebf1*, and *Pax5*, while concomitantly upregulating the myeloid-associated regulator *Cebpa* (Fig. 3A). This transcriptional shift suggests that IL-27 perturbs early lymphoid commitment and favours alternative lineage programmes.

Consistent with this notion, IL-27 enhanced *Cebpa* expression in B-lineage cells under permissive epigenetic conditions, as demonstrated in Reh cells treated with IL-27 in combination with decitabine (Fig. 3B), supporting a potential link between IL-27 signalling and lineage-associated transcriptional reprogramming.

Given that C/EBPα induction has been implicated in lineage redirection at the progenitor level,^45^^,^^46^ we next assessed whether IL-27 affects CLPs in vivo. Exogenous IL-27 substantially reduced both the frequency and absolute number of BM CLPs (Fig. 3C and D). Notably, a comparable reduction was observed in CD19^Cre^;IL-27R^fl/fl^ mice, indicating that IL-27-mediated suppression of CLPs occurs independently of IL-27R signalling in downstream CD19^+^ B-lineage cells.

Consistent with impaired CLP availability, IL-27 treatment reduced total BM B cells and led to relative accumulation of early B-cell progenitors in both WT and CD19^Cre^;IL-27R^fl/fl^ mice (Fig. 3E and F), demonstrating that the developmental blockade is not rescued by B cell-restricted IL-27R deletion.

To evaluate whether IL-27 indirectly inhibits B-cell development via other haematopoietic lineages, we analysed CD4^Cre^;IL-27R^fl/fl^ and Lyz2^Cre^;IL-27R^fl/fl^ mice. Deletion of IL-27R in either compartment failed to restore BM B-cell frequencies or relieve early B-cell progenitors accumulation (Fig. 3G and H), excluding a dominant role for T cell- or myeloid cell-intrinsic IL-27 signalling.

Collectively, these data demonstrate that exogenous IL-27 suppresses CLP formation and disrupts early B-lineage commitment in the BM. This effect is accompanied by *Cebpa* induction and downregulation of lymphoid-associated transcription factors, and occurs independently of IL-27R signalling in mature B cells, T cells, or myeloid cells.

### IL-27 suppresses B-cell development via cell-intrinsic and niche-dependent mechanisms

**Fig. 4:**
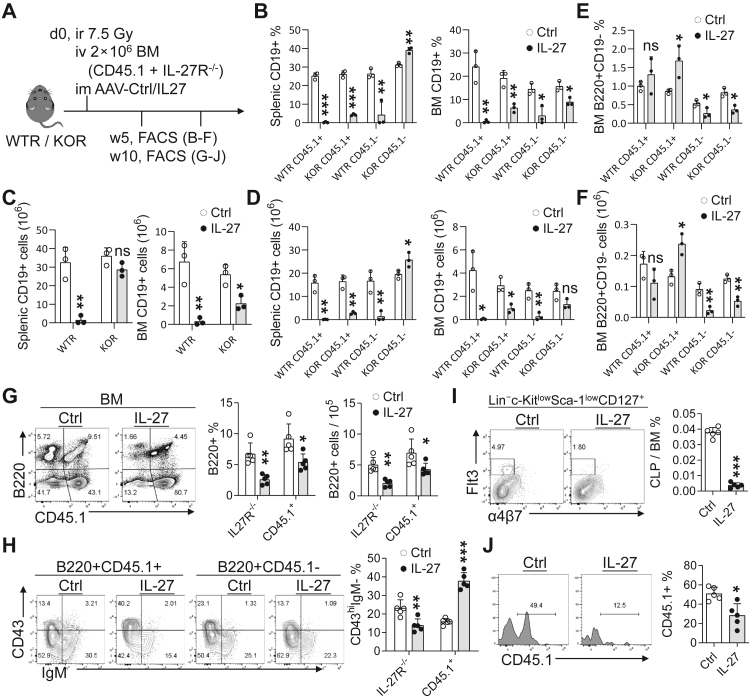
**IL-27 inhibits B-cell development through both direct and indirect mechanisms.** (A) Schematic of the BM chimera model. WT (WTR) or IL-27R^−/−^ (KOR) recipients were treated with AAV-Ctrl or AAV-IL-27. Donor BM cells were a 1:1 mix of WT (CD45.1^+^) and IL-27R^−/−^ (CD45.1^−^) cells. Analyses were performed at 5 weeks (B–E; n = 3 per group) or 10 weeks (F–I; n = 5 per group) post-transplantation. (B) Percentage of CD19^+^ B cells from CD45.1^+^ or CD45.1^−^ (IL-27R^−/−^) donors in spleen and BM of WTR and KOR mice. (C) Absolute numbers of total CD19^+^ B cells in spleen and BM of WTR and KOR mice. (D) Absolute numbers of CD19^+^ B cells from CD45.1^+^ or CD45.1^−^ (IL-27R^−/−^) donors in spleen and BM of WTR and KOR mice. (E) Percentage of B220^+^CD19^−^ B cells from CD45.1^+^ or CD45.1^−^ (IL-27R^−/−^) donors in BM of WTR and KOR mice. (F) Absolute numbers of B220^+^CD19^-^ B cells from CD45.1^+^ or CD45.1^-^ (IL-27R^−/−^) donors in BM of WTR and KOR mice. (G) Frequencies and absolute numbers of BM B220^+^ B cells derived from CD45.1^+^ or IL-27R^−/−^ donors in WTR mice. (H) Proportion of CD43^hi^IgM^−^ subsets from CD45.1^+^ or IL-27R^−/−^ donors in BM B220^+^ B cells in WTR mice. (I–J) Frequencies of CLPs (I) and their donor-specific contributions (J) in BM chimeras. ∗P < 0.05, ∗∗P < 0.01, ∗∗∗P < 0.001; ns, not significant; unpaired Student's t-test.

Building on the observation that IL-27 suppresses CLP formation and early B-lineage commitment, we next employed mixed BM chimeras to distinguish cell-intrinsic from microenvironment-dependent effects. Mixed WT (CD45.1^+^) and IL-27R^−/−^ (CD45.1^−^) donor cells were transplanted into WT (WTR) or IL-27R^−/−^ (KOR) recipients, and B-cell reconstitution was analysed at early and late stages (Fig. 4A).

At the early stage, IL-27 markedly reduced BM B-cell reconstitution in both WTR and KOR recipients (Fig. 4B and C). Donor-tracking analysis revealed that in WTR mice, B cells derived from both CD45.1^+^ WT and CD45.1^−^ IL-27R^−/−^ donors were substantially suppressed by IL-27 in the BM and spleen. In contrast, in KOR recipients, CD45.1^+^ WT donor-derived B cells were markedly reduced in both compartments, whereas IL-27R^−/−^ donor-derived B cells were not suppressed in the BM and spleen (Fig. 4D). These data indicate that IL-27 exerts an indirect inhibitory effect on B cells in WT recipient mice, whereas it requires direct action to suppress B cells in IL-27R^−/−^ recipient mice.

Notably, analysis of the B220^+^CD19^-^ compartment—predominantly pre-pro-B cells—revealed divergent responses. In KOR recipients, CD45.1^+^ donor-derived B220^+^CD19^−^ cells were not suppressed by IL-27 and instead exhibited increases in both frequency and absolute number within the BM, whereas IL-27R^−/−^ donor-derived counterparts were reduced in both WTR and KOR recipients (Fig. 4E and F). These findings indicate that IL-27-mediated suppression of B cells is mechanistically heterogeneous, involving both cell-intrinsic and microenvironment-dependent effects, with subset-specific impacts.

At later stages of reconstitution, IL-27 suppressed B cells derived from both CD45.1^+^ WT and IL-27R^−/−^ donors (Fig. 4G). Consistent with earlier observations, IL-27 induced accumulation of early B-cell progenitors among WT donor-derived BM B220^+^ cells, whereas this effect was attenuated in IL-27R^−/−^ donor-derived counterparts (Fig. 4H), supporting a receptor-dependent block at the pre-pro-B stage.

Given the upstream defects observed above, we next examined CLPs. IL-27 selectively reduced WT donor-derived CLPs, whereas IL-27R^−/−^ donor-derived CLPs were less affected (Fig. 4I and J), further supporting a direct inhibitory effect of IL-27 on WT progenitors.

Collectively, these data demonstrate that IL-27 suppresses B-cell development through dual mechanisms: a direct, IL-27R-dependent inhibition of CLPs and early progenitors, and a concurrent niche-mediated constraint that limits early B-cell output in a recipient- and subset-specific manner.

### Exogenous IL-27 indirectly restrains B-ALL cells in the bone marrow

**Fig. 5:**
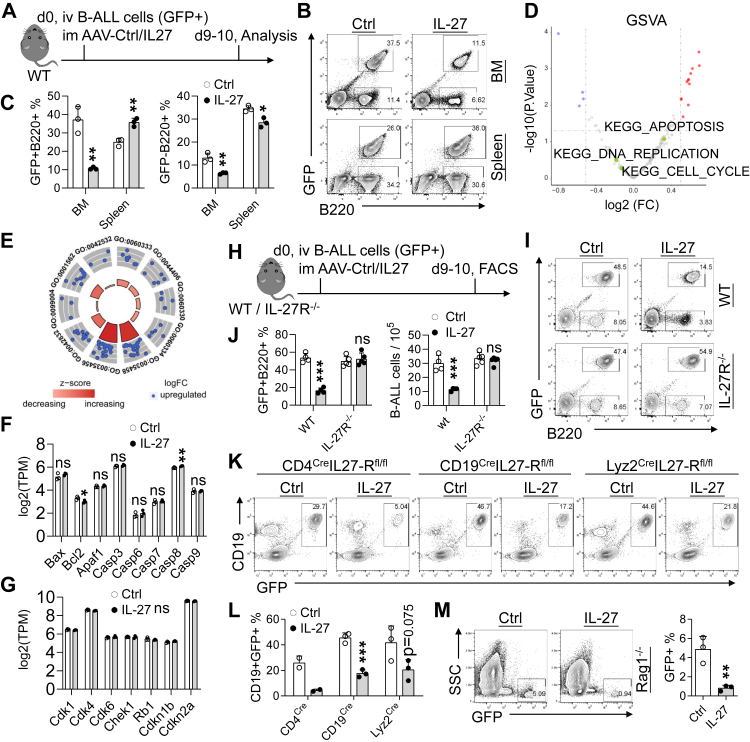
**Exogenous IL-27 indirectly inhibits BM B-ALL cells.** (A) Experimental design. GFP^+^ B-ALL cells were transferred into WT mice, followed by AAV-Ctrl or AAV-IL-27 administration. BM and splenic B-ALL cells were analysed 9–10 days later (n = 3 per group). (B–C) Representative flow cytometry plots and quantification of GFP^+^ B-ALL and GFP^−^ B cells. (D) GSVA analysis of transcriptional changes in BM B-ALL cells isolated from mice in (A). (E) Gene Ontology (GO) enrichment analysis of DEGs between Ctrl and IL-27 groups in (A) (adjusted P < 0.05, |log_2_ fold change| > 1). The outer ring indicates enriched GO terms, the middle ring represents relative gene expression changes in the IL-27 group, and the inner ring shows background gene counts, with colour intensity reflecting enrichment significance. Enriched pathways include interferon-γ-mediated signalling, interferon-β responses, adhesion-related processes, protozoan defence responses, calmodulin-dependent kinase signalling, and negative regulation of STAT tyrosine phosphorylation. (F–G) Expression of apoptosis- and cell cycle-related genes in BM B-ALL cells from (A). (H) Experimental design. WT and IL-27R^−/−^ mice transplanted with GFP^+^ B-ALL cells were treated with AAV-Ctrl or AAV-IL-27 (n = 4 for WT Ctrl group; n = 4 for WT IL-27 group; n = 5 for IL-27R^−/−^ Ctrl group; n = 5 for IL-27R^−/−^ IL-27 group). (I–J) Representative flow plots and quantification of BM B-ALL cells. (K–L) Flow cytometry plots and quantification of BM B-ALL cells in CD4^Cre^;IL-27R^fl/fl^, CD19^Cre^;IL-27R^fl/fl^, and Lyz2^Cre^;IL-27R^fl/fl^ mice (n = 2 for CD4^Cre^ Ctrl group; n = 2 for CD4^Cre^ IL-27 group; n = 3 for CD19^Cre^ Ctrl group; n = 3 for CD19^Cre^ IL-27 group; n = 3 for Lyz2^Cre^ Ctrl group; n = 3 for Lyz2^Cre^ IL-27 group). (M) Flow cytometry plots and quantification of BM B-ALL cells in Rag1^−/−^ mice (n = 3 per group). ∗P < 0.05, ∗∗P < 0.01, ∗∗∗P < 0.001; ns, not significant; unpaired Student's t-test.

Given that IL-27 profoundly disrupts normal B-cell development in the BM, we next investigated whether it similarly affects malignant B cells within the BM niche. In an N-myc-driven B-ALL transfer model, IL-27 treatment markedly reduced the frequency of B-ALL cells in the BM (Fig. 5A–C). In contrast, splenic B-ALL cells were relatively increased, consistent with preferential redistribution rather than systemic leukaemic cell elimination. This pattern mirrors the effects of IL-27 on normal B cells and indicates a preferential restriction of leukaemic cell accumulation within the BM.

Transcriptomic profiling of BM-resident B-ALL cells revealed no enrichment of apoptosis-, cell-cycle-, or DNA replication-related pathways following IL-27 treatment (Fig. 5D and E). Consistently, expression of key apoptosis regulators and cell-cycle genes remained unchanged (Fig. 5F and G), arguing against a direct cytotoxic or cell-intrinsic anti-proliferative effect on B-ALL cells.

To determine whether IL-27 acts directly on B-ALL cells in vivo, leukaemic cells were transplanted into IL-27R^−/−^ recipients. Unlike WT hosts, IL-27 failed to suppress BM B-ALL accumulation in IL-27R^−/−^ mice (Fig. 5I and J), indicating that host IL-27R signalling, rather than leukaemic cell-intrinsic signalling, is required for IL-27-mediated suppression. Notably, conditional deletion of IL-27R in T cells, B cells, or myeloid cells did not appear to eliminate the suppressive effect of IL-27 on BM B-ALL (Fig. 5K–L). Consistently, IL-27 retained antileukaemic activity in Rag1^−/−^ mice lacking adaptive immunity (Fig. 5M).

Together, these data demonstrate that exogenous IL-27 restrains B-ALL accumulation in the BM through an indirect mechanism. Rather than directly killing or arresting leukaemic cells, IL-27 acts in an IL-27R-dependent manner to remodel the BM microenvironment, thereby limiting niche support for both normal and malignant B cells.

### Exogenous IL-27 remodels the bone marrow microenvironment to limit B-cell support and leukaemogenesis

To elucidate the microenvironmental basis for IL-27-mediated indirect suppression of normal and malignant B cells, we analysed global transcriptional changes in the BM following IL-27 exposure, focussing on niche remodelling rather than leukaemic cell-intrinsic effects.

**Fig. 6:**
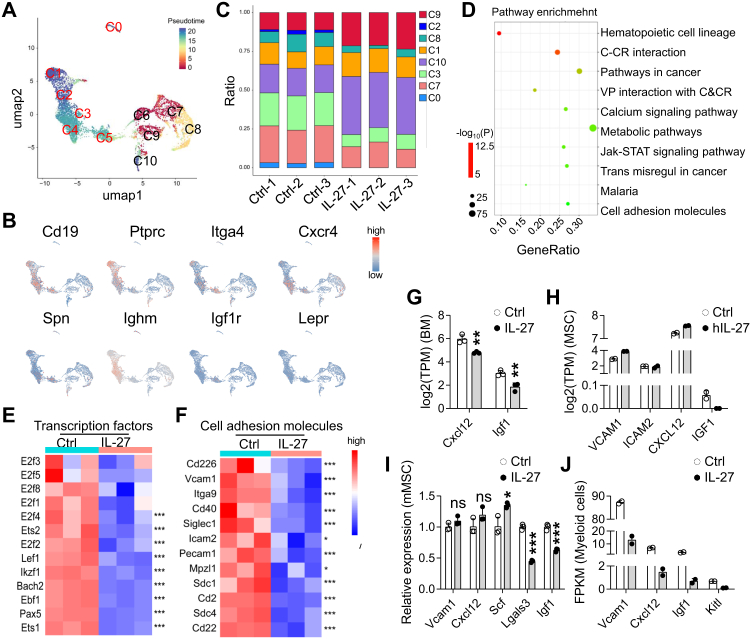
**IL-27 reshapes the BM niche, reducing B-cell support and leukaemogenesis.** (A) Pseudotime analysis of BM B-cell subsets (C0–C10). (B) Expression of representative marker genes across B-cell clusters in (A). (C–G) BM cells were harvested 8 days after AAV-Ctrl or AAV-IL-27 administration for RNA-seq analysis (n = 3 per group). (C) CIBERSORTx analysis showing increased pre-pro-B and cycling pro-B subsets after IL-27 treatment. (D) KEGG pathway enrichment analysis highlighting alterations in adhesion-related pathways. (E–F) Heatmaps of transcription factors (E) and adhesion molecules (F). (G) RNA-seq analysis of genes shown in the figure in BM cells. (H) RNA-seq analysis of genes shown in the figure in human MSCs with or without hIL-27 treatment (n = 2 per group). (I) qPCR analysis of genes shown in the figure in murine MSCs with or without IL-27 (n = 3 per group). (J) RNA-seq analysis of genes shown in the figure in BM Gr-1^+^ cells 3 weeks after AAV-Ctrl or AAV-IL-27 administration (n = 2 per group). ∗P < 0.05, ∗∗P < 0.01, ∗∗∗P < 0.001; ns, not significant; unpaired Student's t-test.

Integration with published scRNA-seq references^41^ and pseudotime reconstruction identified discrete B-cell developmental states within the BM (C0–C10), spanning early progenitors to more mature subsets (Fig. 6A and B). Deconvolution of bulk BM transcriptomes revealed relative accumulation of pre-pro-B (C10) and cycling pro-B (C9) populations after IL-27 treatment (Fig. 6C), consistent with impaired progression beyond early stages rather than increased proliferation.

Pathway enrichment analyses revealed coordinated suppression of cell adhesion and cell–matrix interaction programmes in the BM niche following IL-27 exposure (Fig. 6D). Correspondingly, transcription factors critical for B-cell differentiation (*Ebf1*, *Pax5*, *Ikzf1*, *Bach2*) and adhesion molecules involved in B-cell retention (*Vcam1*, *Icam2*, *Itga9*) were coordinately downregulated (Fig. 6E and F).

In parallel, IL-27 reduced expression of key niche-derived trophic factors. Bulk BM RNA-seq suggested reduced Igf1 expression following IL-27 treatment (Fig. 6G), a pattern similarly observed in both human and murine MSCs treated with IL-27 (Fig. 6H and I). In addition, BM Gr-1^+^ myeloid cells showed decreased expression of several B-cell–supportive factors, including *Vcam1*, *Cxcl12*, *Igf1*, and *Kitl* (Fig. 6J), consistent with IL-27–associated remodelling of multiple non–B-cell compartments within the BM.

Collectively, these data demonstrate that exogenous IL-27 remodels the BM microenvironment by attenuating adhesion cues and trophic support essential for B-cell maintenance. This coordinated niche remodelling provides a mechanistic basis for the indirect suppression of both normal B cells and B-ALL cells, independent of direct effects on B-cell survival or proliferation.

### Exogenous IL-27 enhances therapeutic efficacy and modulates B-cell compartments

**Fig. 7:**
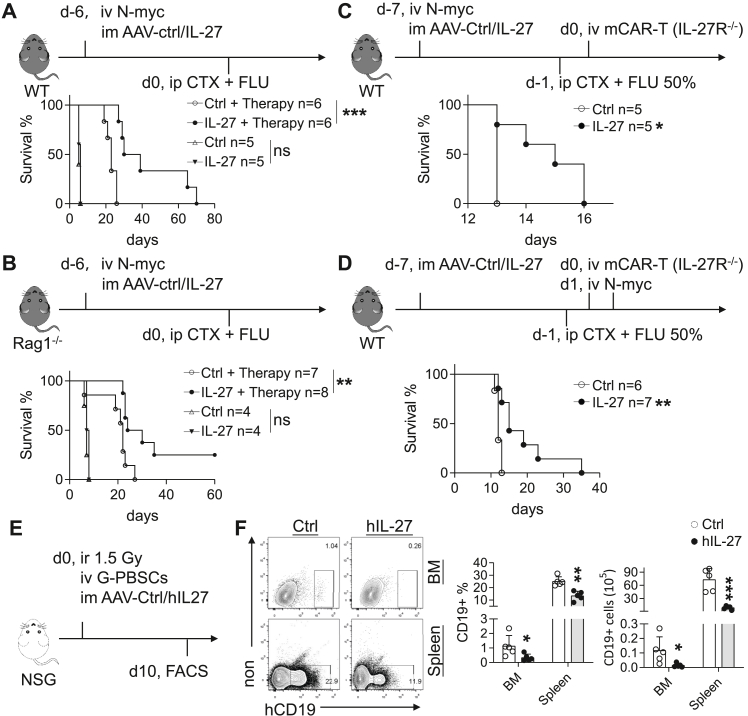
**IL-27 exhibits therapeutic potential while modulating B-cell compartments.** (A–B) Experimental design of combination therapy in WT (A) and Rag1^−/−^ (B) mice, with corresponding Kaplan–Meier survival analysis. (C–D) Experimental design of IL-27R^−/−^ CAR-T cell therapy combined with AAV-Ctrl or AAV-IL-27 in B-ALL-bearing mice, with Kaplan–Meier survival curves. B-ALL cells were transplanted either prior to chemotherapy (C) or after chemotherapy (D). (E–F) NSG mice transplanted with human G-PBSCs were injected intramuscularly with AAV-Ctrl or hIL-27. 10 days later, frequencies and absolute numbers of B cells in the BM and spleen were analysed (n = 5 per group). ∗P < 0.05, ∗∗P < 0.01, ∗∗∗P < 0.001; ns, not significant; log-rank test (A–D) and unpaired Student's t-test (E–F).

To evaluate the therapeutic potential of IL-27 in B-ALL, we first assessed its efficacy in combination with chemotherapy. WT and Rag1^−/−^ mice bearing N-myc-driven B-ALL were treated with AAV-IL-27 or AAV-Ctrl, either alone or alongside standard chemotherapeutic regimens. Kaplan–Meier analyses demonstrated that IL-27 significantly enhanced chemotherapy efficacy in both immunocompetent and lymphocyte-deficient hosts (Fig. 7A and B). These findings indicate that the therapeutic benefit of IL-27 does not require mature adaptive immune cells.

Given that IL-27 enhanced chemotherapy efficacy independently of adaptive immunity, we next asked whether IL-27 could similarly potentiate CAR-T therapy through microenvironmental mechanisms rather than direct effects on CAR-T cells. To dissect the effect of IL-27 on the BM microenvironment rather than its direct action on CAR-T cells, IL-27R^−/−^ CAR-T cells were adoptively transferred into B-ALL-bearing mice post-chemotherapy, in conjunction with AAV-IL-27 or control vectors. IL-27 treatment markedly improved IL-27R^−/−^ CAR-T-mediated antileukaemic efficacy, resulting in prolonged survival (Fig. 7C and D). This enhancement suggests that IL-27 can support cellular immunotherapy, potentially through modulation of the BM microenvironment and/or CAR-T cell fitness.

Finally, to determine whether IL-27 similarly affects human B-cell compartments, NSG mice were reconstituted with human G-PBSCs and treated with AAV-hIL-27 or AAV-Ctrl. hIL-27 significantly reduced both the frequency and absolute number of human B cells in the BM and spleen (Fig. 7E and F), indicating a conserved suppressive effect across species and supporting translational relevance.

Collectively, these data demonstrate that exogenous IL-27 confers therapeutic benefit in B-ALL by enhancing chemotherapy and CAR-T efficacy, while concurrently restraining normal B-cell compartments. This dual activity supports the potential of IL-27 as a complementary strategy for B-cell malignancies.

## Discussion

In this study, we identify exogenous IL-27 as a previously unrecognised regulator of B-cell haematopoiesis and B-ALL progression within the BM. Our data support a dual mechanism whereby IL-27 directly suppresses early B-cell progenitors, particularly CLPs, while concurrently remodelling the BM microenvironment to reduce niche-derived support for both normal and malignant B cells. Together, these findings position IL-27 as a cytokine that links intrinsic progenitor regulation with extrinsic niche control.

A central observation of this study is that IL-27 selectively restrains B-cell reconstitution without globally impairing haematopoietic recovery. This selectivity reflects two coordinated mechanisms. At the progenitor level, IL-27 directly reduces CLP abundance and suppresses early B-lineage differentiation in the BM, accompanied by induction of C/EBPα, a key regulator of early lineage specification. Although the downstream signalling pathways remain to be defined, these data support a direct role for IL-27 in modulating early lymphoid fate decisions. In parallel, IL-27 indirectly limits B-cell output by remodelling the BM niche, as evidenced by reduced expression of critical adhesion molecules and trophic factors, including VCAM-1, ICAM-2, CXCL12 and IGF-1, thereby weakening the microenvironment required for both B-cell development and leukaemic persistence.

Importantly, IL-27-mediated niche remodelling is context- and cell type-dependent. Unlike prior studies performed in isolated cell systems,^47^ our in vivo analyses show that VCAM-1 and CXCL12 are broadly reduced in the BM and in Gr-1^+^ myeloid cells, despite relatively preserved expression in mesenchymal stromal cells, indicating contributions from multiple niche compartments. Among these alterations, suppression of IGF-1 is particularly notable given its established role in B-cell differentiation and leukaemia survival. These findings suggest that IL-27 may modulate metabolic–immune circuits that constrain lymphopoiesis and B-ALL progression in vivo.

Beyond its effects on normal B-cell development, IL-27-driven BM remodelling has important therapeutic implications for B-ALL. The BM niche is a critical determinant of leukaemic survival, chemoresistance and relapse.^48^, ^49^, ^50^ By disrupting niche-derived adhesive and survival signals, IL-27 sensitises B-ALL cells to chemotherapy and limits disease progression. Moreover, IL-27 enhances the efficacy of CAR-T therapy, even when IL-27R^−/−^ CAR-T cells are used, indicating that its therapeutic benefit is largely mediated through microenvironmental remodelling rather than direct effects on effector cells. By reducing both normal B-cell output and leukaemic burden, IL-27 may also alleviate persistent antigen stimulation, a known driver of CAR-T exhaustion, thereby supporting CAR-T persistence and function.

These observations may extend beyond malignancy. Aberrant B-cell development and survival contribute to multiple autoimmune diseases, which are largely treated via peripheral B-cell depletion, with incomplete or transient efficacy.^51^ By targeting early B-cell development while simultaneously weakening BM niche support, IL-27 may represent a complementary strategy for B-cell-mediated disorders, pending further validation.

In summary, our study establishes IL-27 as a regulator of B-cell haematopoiesis and B-ALL progression through coordinated inhibition of CLPs and remodelling of the BM niche. These findings advance our understanding of cytokine–niche interactions and provide a conceptual framework for exploiting IL-27 to enhance chemotherapy and cellular immunotherapies while overcoming niche-mediated resistance.

### Limitations of the study

C/EBPα is known to influence lineage decisions of CLPs and B cells. While IL-27 upregulates C/EBPα and suppresses CLPs, the precise mechanisms underlying this regulation, including potential contributions from C/EBPα and other downstream pathways, remain to be fully clarified. Similarly, the broader effects of IL-27 on individual BM niche cells and their role in B-cell modulation warrant further study.

Safety and immune homoeostasis remain key considerations for IL-27 therapies. While long-term AAV-IL-27 did not induce overt autoimmunity in mice,^16^^,^^17^ careful evaluation of immune skewing, infection risk, and organ toxicity is required. Targeted delivery to the BM may help mitigate potential side effects.

## Contributors

JMZ, XFB, and CWD designed the study and drafted the manuscript with input from all authors; JMZ and CWD accessed and verified the underlying data. JMZ performed most experiments, JX analysed RNA-seq data and validated transgenic mice, KMC provided technical guidance, and all authors approved the final manuscript.

## Data sharing statement

Data supporting the findings of this study will be made available upon reasonable request, beginning at publication and with no end date. Proposals should be directed to zhu-jianmin@scmc.com.cn.

## Declaration of interests

The authors declare no competing financial or non-financial interests.

## Appendix A Supplementary data

Supplementary Table 1

Reagent Validation
