# Supplementary material for: Interleukin-27 remodels the bone marrow niche to suppress B-cell development and leukaemia progression in mouse models
Source: eBioMedicine. 2026 Apr 2;127:106239. doi: 10.1016/j.ebiom.2026.106239 (PMC13085022; doi:10.1016/j.ebiom.2026.106239)
Supplement: Supplementary Table 1 [file mmc1.docx]

**Supplementary Table 1. Antibodies used in FACS analysis**

| Antibody | Clone | Catalog | Vendor | RRID |
| --- | --- | --- | --- | --- |
| B220-APCcy7 | RA3-6B2 | 103224 | BioLegend | RRID:AB_313007 |
| B220-FITC | RA3-6B2 | 103206 | BioLegend | RRID:AB_312991 |
| B220-PE | RA3-6B2 | 103208 | BioLegend | RRID:AB_312993 |
| Sca-1-APCcy7 | D7 | 108126 | BioLegend | RRID:AB_10645327 |
| Flt3-PE | A2F10 | 135306 | BioLegend | RRID:AB_1877217 |
| CD11b-APC | M1/70 | 561690 | BD Biosciences | RRID:AB_10897015 |
| CD3-APC | 17A2 | 565643 | BD Biosciences | RRID:AB_2739319 |
| NK1.1-PE | PK136 | 561046 | BD Biosciences | RRID:AB_10563766 |
| hCD19-APC | HIB19 | 555413 | BD Biosciences | RRID:AB_395813 |
| CD45-SB600 | 30-F11 | 63-0451-82 | eBioscience | RRID:AB_2637149 |
| CD19-APC | eBio1D3 (1D3) | 17-0193-82 | eBioscience | RRID:AB_1659676 |
| CD19-PE | eBio1D3 (1D3) | 12-0193-82 | eBioscience | RRID:AB_657659 |
| CD19-FITC | eBio1D3 (1D3) | 11-0193-82 | eBioscience | RRID:AB_657666 |
| IgM-APCef780 | II/41 | 47-5790-82 | eBioscience | RRID:AB_2573984 |
| CD43-PEcy7 | eBioR2/60 | 11-0431-82 | eBioscience | RRID:AB_465040 |
| Gr-1-ef450 | RB6-8C5 | 48-5931-82 | eBioscience | RRID:AB_1548788 |
| F4/80-PE | BM8 | 12-4801-82 | eBioscience | RRID:AB_465923 |
| CD4-PEcy7 | GK1.5 | 25-0041-82 | eBioscience | RRID:AB_469576 |
| Foxp3-PE | FJK-16s | 12-5773-82 | eBioscience | RRID:AB_465936 |
| CD25-APC | PC61.5 | 17-0251-82 | eBioscience | RRID:AB_469366 |
| c-Kit-BV605 | 2B8 | 406-1171-82 | eBioscience | RRID:AB_3666070 |
| CD127-PEcy7 | A7R34 | 25-1271-82 | eBioscience | RRID:AB_469649 |
| α4β7-APC | DATK32 | 17-5887-82 | eBioscience | RRID:AB_1210577 |
| CD45.1-PE | A20 | 12-0453-82 | eBioscience | RRID:AB_465675 |
| CD45.1-FITC | A20 | 11-0453-82 | eBioscience | RRID:AB_465058 |
| CD45.1-APCef780 | A20 | 47-0453-82 | eBioscience | RRID:AB_1582228 |
| Lin-ef450 | 17A2(CD3)、RA3-6B2(B220)、M1/70(CD11b)、TER-119(TER-119)、RB6-8C5(Gr-1) | 88-7772-72 | eBioscience | RRID:AB_10426799 |
