# Supplementary material for: Interleukin-27 remodels the bone marrow niche to suppress B-cell development and leukaemia progression in mouse models
Source: eBioMedicine. 2026 Apr 2;127:106239. doi: 10.1016/j.ebiom.2026.106239 (PMC13085022; doi:10.1016/j.ebiom.2026.106239)
Supplement: Reagent Validation [file mmc2.pdf]

Supplemental Data: Reagent Validation – STR Authentication for Cell Lines

Only the Reh cell line was used in this study; the STR profiles of other tested cell lines (NALM-6, MCF 10A) are provided for completeness and not relevant to the present research.

STR Authentication Report for Reh Cell Line

Testing Institution: Shanghai Biowing Applied Biotechnology Co., Ltd.

Submitting Institution: Shanghai Children's Medical Center

Report Date: July 25, 2019

Sample Information for Reh: Client sample ID = R; Internal laboratory ID = 20190724-01

Detection Method: Genomic DNA was extracted using the Axygen genomic DNA extraction kit. Short tandem repeat (STR) genotyping was performed via a **21-STR amplification protocol**, and the STR loci plus the sex-determining Amelogenin locus were analyzed on an ABI 3730XL Genetic Analyzer. STR profile matching was conducted against the DSMZ cell line database (integrating STR data from ATCC, DSMZ, JCRB and RIKEN databases).

Key Validation Results for Reh Cell Line:

1. No multi-allelic loci (≥3 alleles) were detected in the Reh cell line, indicating a normal STR genotyping profile.
2. The STR profile of the tested Reh cell line showed a **basic match** with the reference Reh cell line (DSMZ, CRL-8286) with a matching score (EV value) of 0.94.
3. All aligned STR loci (D5S818, D13S317, D7S820, D16S539, VWA, TH01, AMEL, TPOX) exhibited **100% concordance** in allele sizes with the DSMZ reference. A single locus discrepancy was observed at CSF1PO: the tested Reh cell line had alleles 11/12, while the DSMZ reference had alleles 10/11.
4. Additional STR loci (D12S391, FGA, D2S1338, etc.) were successfully amplified and genotyped for the tested Reh cell line with clear, interpretable results.

Note: This document is the original Chinese STR authentication report with complete genotyping data for the Reh cell line (and other tested cell lines) attached in the following pages. All experimental procedures and STR profiling comply with standard cell line authentication guidelines recommended by the International Cell Line Authentication Committee (ICLAC).

细胞遗传质量鉴定检测

Cell Line Authentication Service

STR 基因型检测报告

送检单位：上海儿童医学中心

检品名称：细胞系

委托单位：上海翼和应用生物技术有限公司

报告日期：2019-07-25

上海翼和应用生物技术有限公司　联系地址：上海市松江区龙腾路 1015 弄中星创意园 2 号 502 室
网址：http://www.biowing.com.cn/邮箱：market@biowing.com.cn　服务热线：021-33559491

报 告 说 明

1. 本报告只对送检的来样负责。
2. 检验报告上的检验结果和检验单位名称，未经同意不得用于广告、评优及商业宣传。
3. 对本报告有异议，请于收到报告之日起十五日内以书面方式提出，逾期不予受理。
4. 对纸质检验报告涂改、增删，或未加盖检验单位印章的复印件均无效。

样 品 信 息

样品编号：

客户样本编号	公司编号
R	20190724-01
N	20190724-02
10A	20190724-03

样品数量： 3

样品性状： 细胞系

检测项目： STR

送检单位： **上海儿童医学中心**

检测方法： 用 Axygen 的基因组抽提试剂盒提取 DNA，采用 21- STR 扩增方案扩增，在 ABI 3730XL 型遗传分析仪上对 STR 位点和性别基因 Amelogenin 进行检测。

上海翼和应用生物技术有限公司 联系地址：上海市松江区龙腾路 1015 弄中星创意园 2 号 502 室
网址：http://www.biowing.com.cn/邮箱：market@biowing.com.cn 服务热线：021-33559491

检 测 结 果

(一)　检验基本情况

公司编号	多等位基因	匹配细胞系	细胞库	EV 值	匹配说明
20190724-01	无	Reh	DSMZ	0.94	基本匹配
20190724-02	有	NALM-6	DSMZ	0.90	基本匹配
20190724-03	无	MCF 10A	DSMZ	1.00	完全匹配

样本基因型检验结果

- 多等位基因指三等位及以上基因现象。
- 本次检测各细胞分型结果良好。

(二)　各样本描述

- 20190724-01：该株细胞 DNA 分型在细胞系检索中找到**基本匹配**的细胞系，DSMZ 数据库显示细胞名为 **Reh**，细胞号对应 **CRL-8286**。本次检测在该细胞系中**没有发现多等位基因**。
- 20190724-02：该株细胞 DNA 分型在细胞系检索中找到**基本匹配**的细胞系，DSMZ 数据库显示细胞名为 **NALM-6**，细胞号对应 **128**。本次检测在该细胞系中**发现多等位基因**。
- 20190724-03：该株细胞 DNA 分型在细胞系检索中找到**完全匹配**的细胞系，DSMZ 数据库显示细胞名为 **MCF 10A**，细胞号对应 **CRL-10317**。本次检测在该细胞系中**没有发现多等位基因**。

备注：待测细胞系与收录于 ATCC, DSMZ, JCRB 和 RIKEN 数据库的细胞系 STR 数据进行比对，未收录于以上细胞库的细胞系将无法匹配。

（三）样本分型结果

细胞的 STR 位点和 Amelogenin 位点的基因分型结果						
Loci	送检细胞 STR 信息			细胞库细胞 STR 信息		
	送检细胞名：R			细胞库细胞名：Reh		
	Allele1	Allele2	Allele3	Allele1	Allele2	Allele3
D5S818	11	13		11	13	
D13S317	11	13		11	13	
D7S820	9	12		9	12	
D16S539	9	9		9	9	
VWA	14	15		14	15	
TH01	7	7		7	7	
AMEL	X	X		X	X	
TPOX	8	8		8	8	
CSF1PO	11	12		10	11	
D12S391	23	25				
FGA	22	23				
D2S1338	20	22				
D21S11	28	30				
D18S51	10	14				
D8S1179	13	15				
D3S1358	18.2	18.2				
D6S1043	11	21				
PENTAE	7	11				
D19S433	12	14				
PENTAD	9	11				
D1S1656	15	15				

上海翼和应用生物技术有限公司 联系地址：上海市松江区龙腾路 1015 弄中星创意园 2 号 502 室
网址：http://www.biowing.com.cn/邮箱：market@biowing.com.cn 服务热线：021-33559491

细胞的 STR 位点和 Amelogenin 位点的基因分型结果						
Loci	送检细胞 STR 信息			细胞库细胞 STR 信息		
	送检细胞名：N			细胞库细胞名：NALM-6		
	Allele1	Allele2	Allele3	Allele1	Allele2	Allele3
D5S818	11	12		11	12	10
D13S317	9	12		9	12	8
D7S820	8	10		8	10	9
D16S539	10	11		10	11	9
VWA	15	16		15	16	
TH01	8	9		8	9	
AMEL	X	Y		X	Y	
TPOX	8	10		8	10	
CSF1PO	12	12		12	12	
D12S391	20	21				
FGA	22	23				
D2S1338	17	24				
D21S11	28	29				
D18S51	12	15				
D8S1179	12	13				
D3S1358	16	17				
D6S1043	10	11	12			
PENTAE	11	11				
D19S433	13	15				
PENTAD	8	14				
D1S1656	11	17				

细胞的 STR 位点和 Amelogenin 位点的基因分型结果						
Loci	送检细胞 STR 信息			细胞库细胞 STR 信息		
	送检细胞名：10A			细胞库细胞名：MCF 10A		
	Allele1	Allele2	Allele3	Allele1	Allele2	Allele3
D5S818	10	13		10	13	
D13S317	8	9		8	9	
D7S820	10	11		10	11	
D16S539	11	12		11	12	
VWA	15	17		15	17	
TH01	8	9.3		8	9.3	
AMEL	X	X		X	X	
TPOX	9	11		9	11	
CSF1PO	10	12		10	12	
D12S391	17	20				
FGA	22	24				
D2S1338	21	26				
D21S11	28	30				
D18S51	18	19				
D8S1179	14	16				
D3S1358	14	18				
D6S1043	12	18				
PENTAE	13	14				
D19S433	13	15				
PENTAD	10	12				
D1S1656	17	17.3				

上海翼和应用生物技术有限公司 联系地址：上海市松江区龙腾路 1015 弄中星创意园 2 号 502 室
网址：http://www.biowing.com.cn/邮箱：market@biowing.com.cn 服务热线：021-33559491

其他说明

(一) 分型方案及位点分布

	方案 1	方案 2	方案 3	方案 4
1	D3S1358	D8S1179	D19S433	AMEL
2	VWA	D21S11	TH01	D1S1656
3	D7S820	D16S539	D13S317	D5S818
4	CSF1PO	D2S1338	TPOX	D12S391
5	PENTAE	PENTAD	D18S51	FGA
6			D6S1043	

实验方案及位点

(二) STR 数据库比对

本公司采用 DSMZ tools 进行细胞系比对，其中包含来自于 ATCC, DSMZ, JCRB 和 RIKEN 数据库的 2455 个细胞系 STR 数据。如果待检测细胞未收录于以上细胞库或这是自行建立的新细胞系将无法进行比对，用户需根据细胞分型结果自行与其他数据库进行比对。

(三) 文献引用参考

1．Authentication testing of HEK 293T and HeLa cell lines have been performed by Shanghai Biowing Applied Biotechnology Co.,Ltd via STR profiling. STR profiles match the standards recommended for HEK 293T and HeLa cell lines authentication

2．AGS, NCI-N87, HGC-27 and HEK293 were STR-authenticated on Dec. 8, 2015 by Shanghai Biowing Applied Biotechnology Co. LTD, Shanghai, China

主要实验人员：张佳男

复核人: 钱宁

负责人：白杨

签发日期：2019-07-25

上海翼和应用生物技术有限公司 联系地址：上海市松江区龙腾路 1015 弄中星创意园 2 号 502 室
网址：http://www.biowing.com.cn/邮箱：market@biowing.com.cn 服务热线：021-33559491
